# Supplementary material for: Myeloid cell nuclear differentiation antigen controls the pathogen-stimulated type I interferon cascade in human monocytes by transcriptional regulation of IRF7
Source: Nat Commun. 2022 Jan 10;13:14. doi: 10.1038/s41467-021-27701-x (PMC8748983; doi:10.1038/s41467-021-27701-x)
Supplement: Supplementary file 1 — Supplementary Information [file 41467_2021_27701_MOESM1_ESM.pdf]

Supplementary information

Myeloid cell nuclear differentiation antigen controls the pathogen-stimulated type I interferon casca de in human monocytes by transcriptional regulation of IRF7

Lili Gu[1,6], David Casserly[1,6], Gareth Brady[2], Susan Carpenter[3], Adrian P. Bracken[4], Katherine A Fitzgerald[3], Leonie Unterholzner[1,5] and Andrew G Bowie[1,7*]

[1]School of Biochemistry and Immunology, Trinity Biomedical Sciences Institute and [2]School of Medicine, Trinity College Dublin, Dublin 2, Ireland. [3]Division of Infectious Diseases and Immunology, University of Massachusetts Medical School, Worcester, Massachusetts, USA. [4]Smurfit Institute of Genetics, Trinity College Dublin, Dublin 2, Ireland. [5]Current address: Division of Biomedical and Life Sciences, Faculty of Health and Medicine, Lancaster University, Lancaster LA1 4YQ, UK.
[6]These authors contributed equally
[7]Lead Contact
[*]Correspondence: agbowie@tcd.ie

Supplementary Table 1: List of primers used for quantitative RT-PCR

Target	Sequence (5'---3')
β-actin (forward)	CGCGAGAGAAGATGACCCAGATC
β-actin (reverse)	GCCAGAGGCGTACAGGGATA
MNDA (forward)	TACTCCGAATCAGGAAACCCAGGCC
MNDA (reverse)	TGGCGCTGTTGCTTTCAGTACCAA
IFI16 (forward)	CCGTTCATGACCAGCATAGG
IFI16 (reverse)	TCAGTCTTGGTTTCAACGTGGT
AIM2 (forward)	TGGGCATGCTCTCCTGAGTCCTC
AIM2 (reverse)	TGACAACTTTGGGATCAGCCTCCTG
PYHIN1 (forward)	TCACCAAGAAGGATGAAACCC
PYHIN1 (reverse)	CGGTTGGTGGAAGTGTCAGA
IFNβ (forward)	CTGCATTACCTGAAGGCCAAG
IFNβ (reverse)	TTGAAGCAATTGTCCAGTCCC
IFNα (forward)*	TGAAGGACAGACATGACTTTGG
IFNα (reverse)*	TCCTTTGTCCTGAAGAGATTGA
IFNα14 (forward)	TGAATTTCCCCAGGAGGAA
IFNα14 (reverse)	TCCCAAGCAGCAGATGAGTT
IRF7 (forward)	AGCGGCTGCTATGAGGGGCT
IRF7 (reverse)	GCCACAGCCCAGGCCTTGAA
IFIT1 (forward)	CACCATTGGCTGCTGTTTAGCTCC
IFIT1 (reverse)	GGCAGCCGTTCTGCAGGGTTTT
IFIT2 (forward)	TTCTCCCTCCATCAAGTTCCAG
IFIT2 (reverse)	GCACTGCAACCATGAGTGAGAA
IRF1 (forward)	AGGCCAAGAGGAAGTCATGTG
IRF1 (reverse)	CTGTGTAGCTGCTGTGGTCA
ISG15 (forward)	TTTGCCAGTACAGGAGCTTGTG

ISG15 (reverse)	GGGTGATCTGCGCCTTCA
IRF3 (forward)	CTGGGGCCCTTCATTGTAGA
IRF3 (reverse)	GCACAACCTTGACCATCACG
IRF5 (forward)	AAGCCATCCGGCCAA
IRF5 (reverse)	GGAAGTCCCGGCTCTTGTTAA
IFNλ−1 (forward)	CTGCCACATTGGCAGGTTCA
IFN λ−1 (reverse)	AGACAGGAGAGCTGCAACTC
IFN λ−2 (forward)	AGTCGCTTCTGCTGAAGGAC
IFN λ−2 (reverse)	TCCAGAACCTTCAGCGTCAG

*IFNα primers were designed to target transcripts for IFNα2, IFNα5 and IFNα21.

Supplementary Table 2: List of primers used for chromatin immunoprecipitation

**Target**	**Sequence (5'---3')**
IFNα14 ChIP (forward)	AAGCCCATGGGGCAGGGAA
IFNα14 ChIP (reverse)	GGGCTGGTTGATGAGGGGT
IFNβ ChIP (forward)	TAGTCATTCACTGAAACTTTA
IFNβ ChIP (reverse)	AGGTTGCAGTTAGAATGTC
IRF7 ChIP (forward)	ATCTTGCGCCAAGACAATTCAGGG
IRF7 ChIP (reverse)	TTGTGGCACTGCTCACCAGTAGAT
IRF7 promoter -1000 (forward)	ACCCTACACAGAGATTGCCT
IRF7 promoter -865 (reverse)	CTCCAACCCAAGCATGTAGATT
IRF7 promoter -900(forward)	TAGTCCCCTTCTGAAATCTACATG
IRF7 promoter -767 (reverse)	CCTAGGCCTCCTGGTCT
IRF7 promoter -800 (forward)	GCCTGAGCAGGAGCCA
IRF7 promoter -660 (reverse)	TTAGGGAAAGTGCTGACAAGCC
IRF7 promoter -700 (forward)	CATCAAGGGATCCCTTGCG

IRF7 promoter -538 (reverse)	GCTGGGATTTAGGCGAGAG
IRF7 promoter -600 (forward)	CGGGAGGCTTAGGTAGGA
IRF7 promoter -440 (reverse)	GTGGACGTGCCTCGAAA
IRF7 promoter -500 (forward)	GGAGTTCGACACCAGCC
IRF7 promoter -384 (reverse)	CTACCTAAGCCTCCCGAGTA
IRF7 promoter -300 (forward)	CTCCATCTCAAAAAAAGAAACGC
IRF7 promoter -189 (reverse)	GGGACAGAGCAAGACTCAG
IRF7 promoter -200 (forward)	TGCTCTGTCCCCTGGGCT
IRF7 promoter -83 (reverse)	ATGCGACCAGCGGAACC
IRF7 promoter -100 (forward)	GGTTCCGCTGGTCGCAT
IRF7 promoter +20 (reverse)	GGACGGGAAGTTTCGTCTCG
IRF7 promoter 0 (forward)	CGAGACGAAACTTCCCGT
IRF7 promoter +132 (reverse)	CCCAGCTCTTGGCTCTAC
IRF7 promoter +200 (forward)	AACCGGACGGGGTGGGAT
IRF7 promoter +353 (reverse)	CAGGTGTTGAACCAGTGTCCAG
IRF7 promoter +350 (forward)	AACCGGACGGGGTGGGAT
IRF7 promoter +488(reverse)	CAGGTGTTGAACCAGTGTCCAG
IRF7 promoter +450 (forward)	CCCTCTGCCAAGAGATCCATA
IRF7 promoter +570 (reverse)	GGAGGTAAGGGCTCCTGT
IRF7 promoter +570 (forward)	ACAGGAGCCCTTACCTCC
IRF7 promoter +671 (reverse)	TGGCATCTGGAGAGGGT
EIF4A2 Promoter (forward)	TGGTGTCATCGAGAGCAACTG
EIF4A2 Promoter (reverse)	GGCTTCTCAAAACCGTAAGCA
SAT2 promoter (forward)	CATCGAATGGAAATGAAAGGAGTC
SAT2 promoter (reverse)	ACCATTGGATGATTGCAGTCAA
IRF1 promoter (forward)	CTCCATCTCAAAAAAAGAAACGC
IRF1 promoter (reverse)	GGGACAGAGCAAGACTCAG

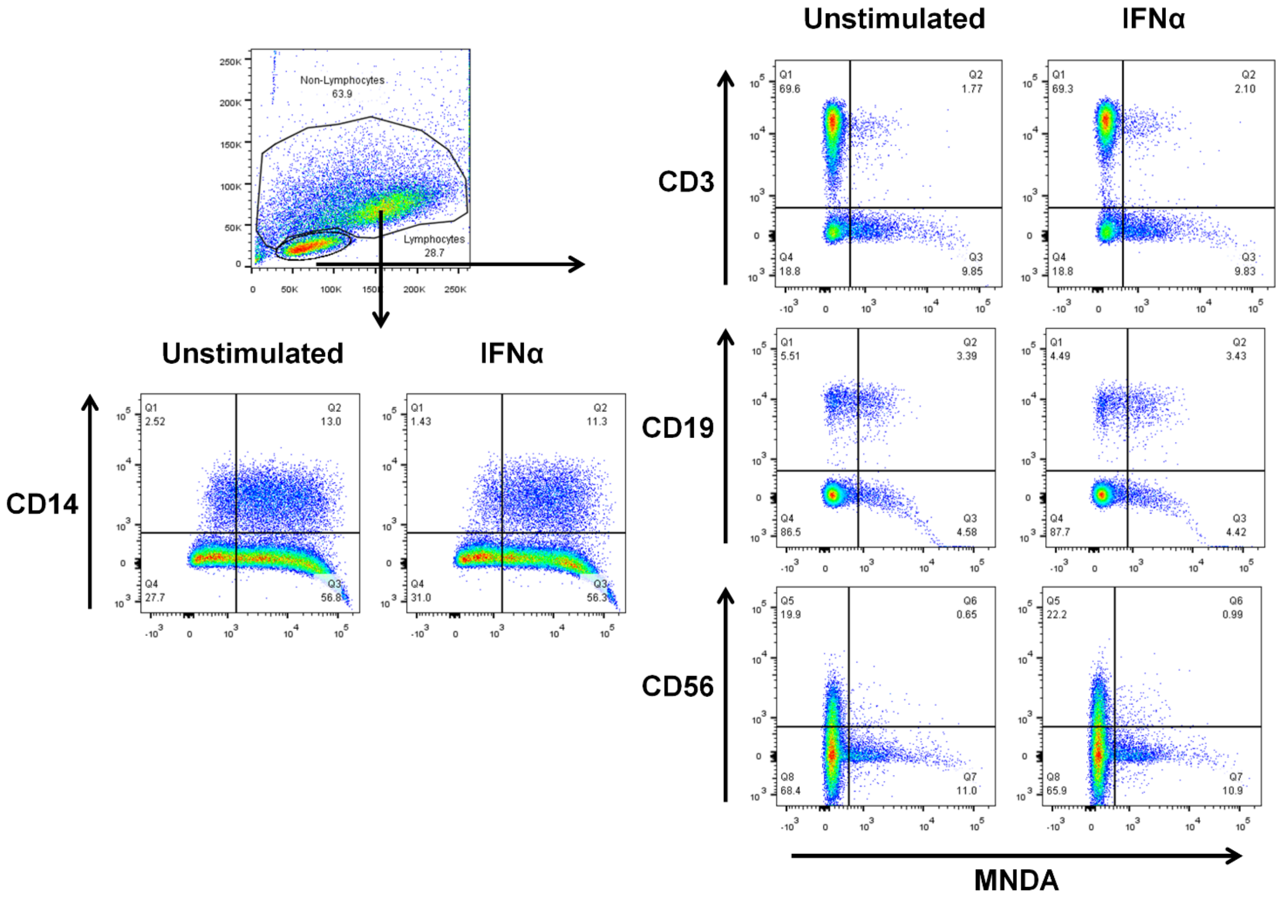

Supplementary Figure 1. MNDA is expressed in monocytes and B cells in PBMC sub-populations. Human peripheral blood mononuclear cells (PBMCs) isolated from buffy coats were stimulated with 1000 u/ml of IFNα for 16 h and prior to staining with the indicated antibodies. Cells were gated on lymphocytes for the lymphoid markers CD3 (for T cells), CD19 (for B cells) and CD56 (for NK cells), and the non-lymphocyte cells were gated for the myeloid marker CD14 (for monocytes). Plots show that MNDA is primarily expressed in CD14+ monocytes and CD19+ B cells. Representative of three independent experiments.

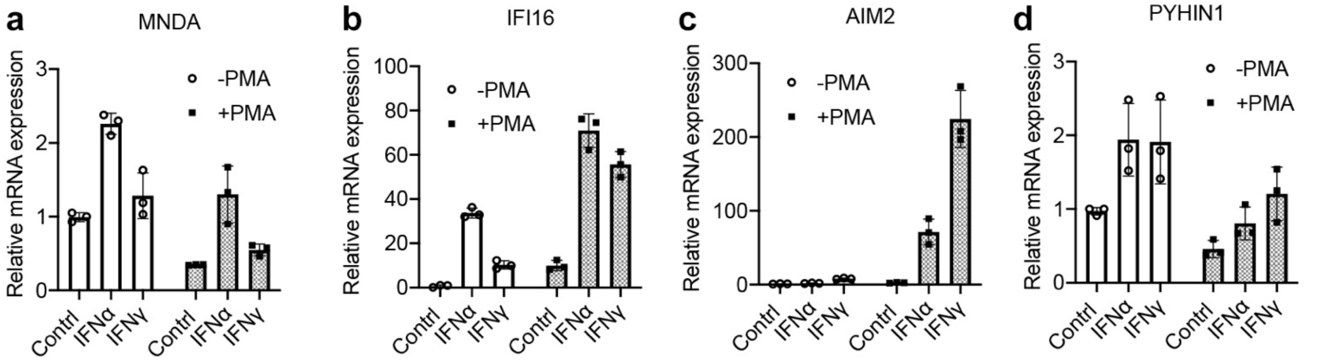

Supplementary Figure 2. Expression of MNDA and other PYHIN proteins in THP-1 cells. (a-d) Quantitative PCR analysis of mRNA expression of MNDA (a), IFI16 (b), AIM2 (c) or PYHIN1 (d) in unprimed (-PMA) or PMA primed (+PMA) THP-1 cells treated with IFNα (1000 U/ml) or IFNγ (50 ng/ml) for 6 h. Data are mean ± SD of triplicate samples and are representative of three independent experiments.

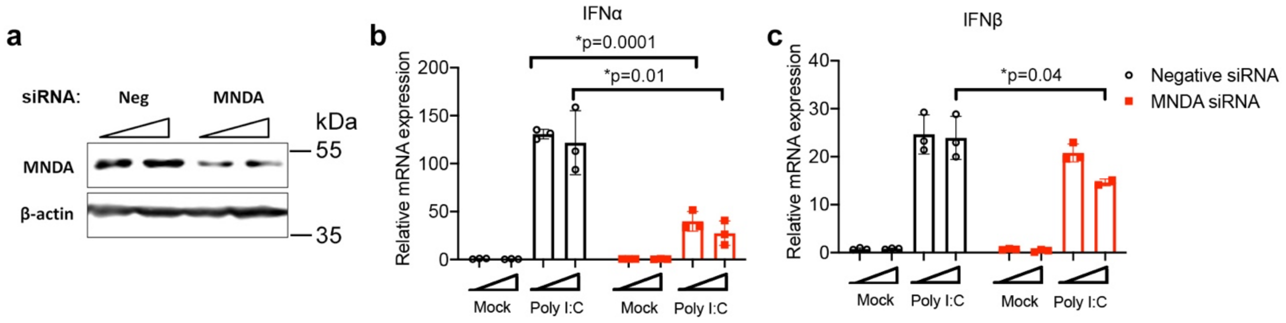

Supplementary Figure 3. MNDA siRNA supresses RNA-stimulated type I IFN induction (a) Immunoblot analysis of MNDA protein expression in THP-1 cells electroporated with 50 and 100pmol (wedges) of negative control siRNA or MNDA siRNA for 48 h. Representative of three experiments. (b, c) Quantitative PCR analysis of mRNA expression of IFNα (b) or IFNβ (c) from THP-1 cells electroporated with 50 and 100 pmol (wedges) of negative control siRNA or MNDA siRNA for 24 h, prior to transfection with poly(I:C) for a further 24 h. Data are mean ± SD of triplicate samples and are representative of three independent experiments; two tailed unpaired Students *t*-test; *p<0.05 indicates significance compared to respective groups.

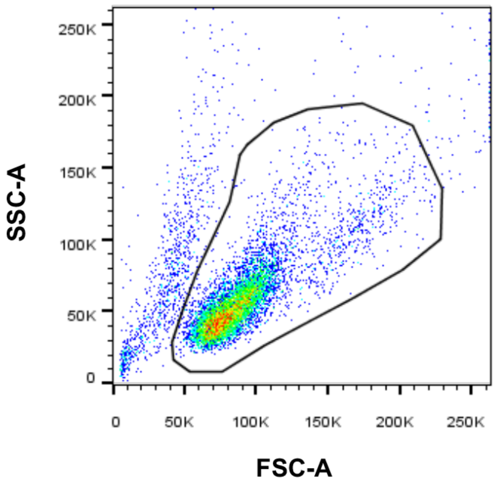

Supplementary Figure 4. Gating strategy for detection of VSV-GFP in THP-1 cells. Flow cytometry analysis showing gating strategy for the sorting of GFP-positive cells, using THP-1 cells stably expressing control shRNA and infected with VSV-GFP. Related to Fig. 2e.

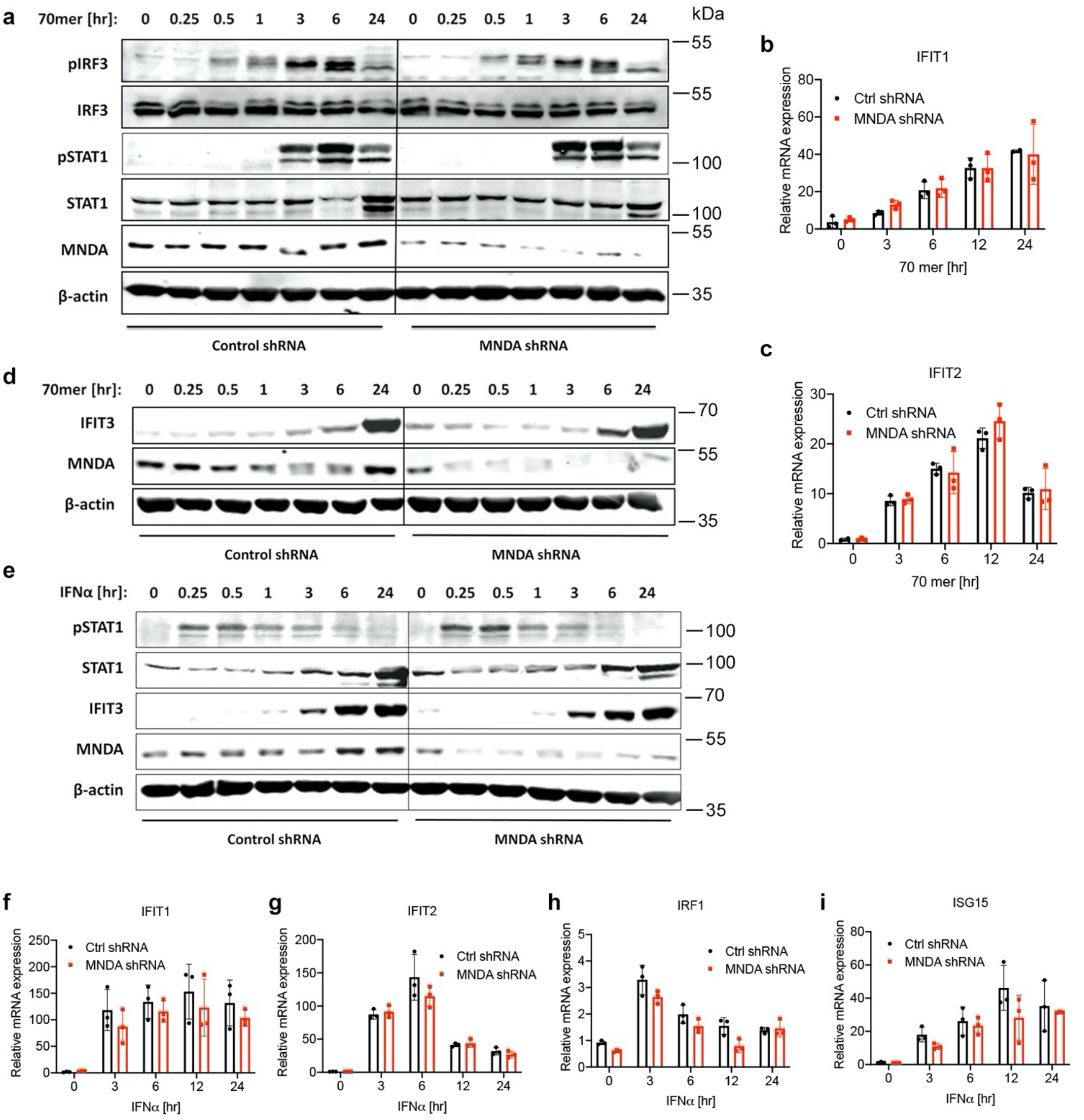

Supplementary Figure 5. Normal activation of PRR and IFNAR signalling in cells with reduced MNDA expression. (a) THP-1 cells expressing control or MNDA shRNA were transfected with dsVACV 70mer (1 µg/ml) for the indicated times and cell lysates prepared and immunoblotted for the indicated proteins. (b, c) Quantitative PCR analysis of mRNA expression of IFIT1 (b) or IFIT2 (c) from THP-1 cells expressing control or MNDA shRNA and transfected with dsVACV 70mer (1 µg/ml) for the indicated times. (d) Cell were treated as in (a) and the indicated proteins detected by immunoblot. (e) THP-1 cells expressing control or MNDA shRNA were stimulated with IFNα (1000 U/ml) for the indicated times and cell lysates prepared and immunoblotted for the indicated proteins. (f-i) Quantitative PCR analysis of mRNA expression of IFIT1 (f), IFIT2 (g), IRF1 (h) or ISG15 (i) from THP-1 cells expressing control or MNDA shRNA and stimulated with IFNα (1000 U/ml) for the indicated times. All data are mean ± SD of triplicate samples and are representative of three independent experiments. Immunoblots (a, d, e) are representative of three experiments.

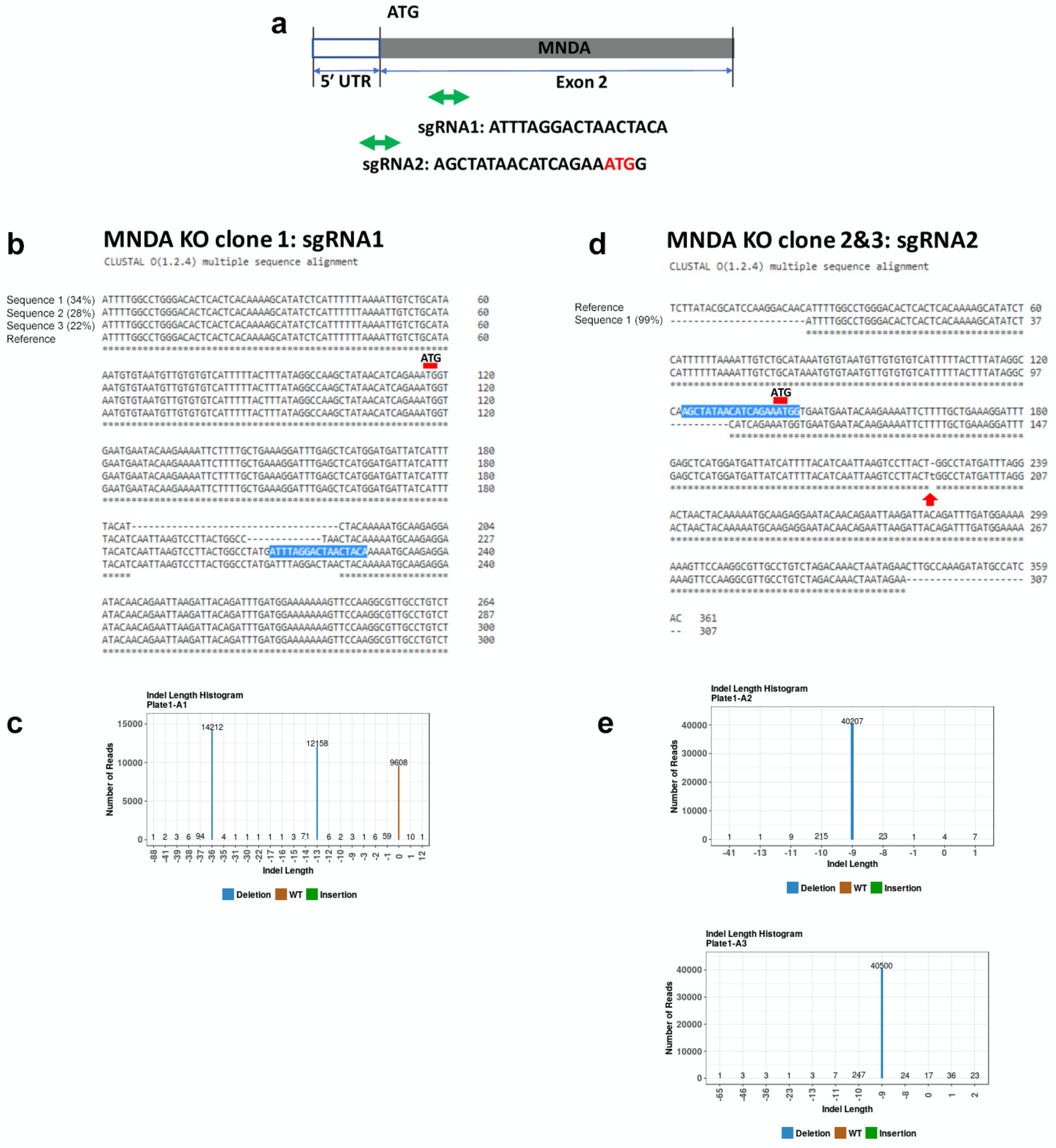

Supplementary Figure 6. Genetic ablation of MNDA expression in THP-1 cells. (a) Schematic diagram of human MNDA-specific sgRNA1 and sgRNA2 used in CRISPR/Cas9 gene editing of *MNDA* locus. (b, d) Sequence alignment of MNDA locus in MNDA$^{-/-}$ clones 1-3. (c, e) Indel length histograms from next generation sequences (NGS) analysis shows disruption of MNDA alleles in *MNDA*$^{-/-}$ clones 1 (c) or 2 (e, upper panel) and 3 (e, lower panel).

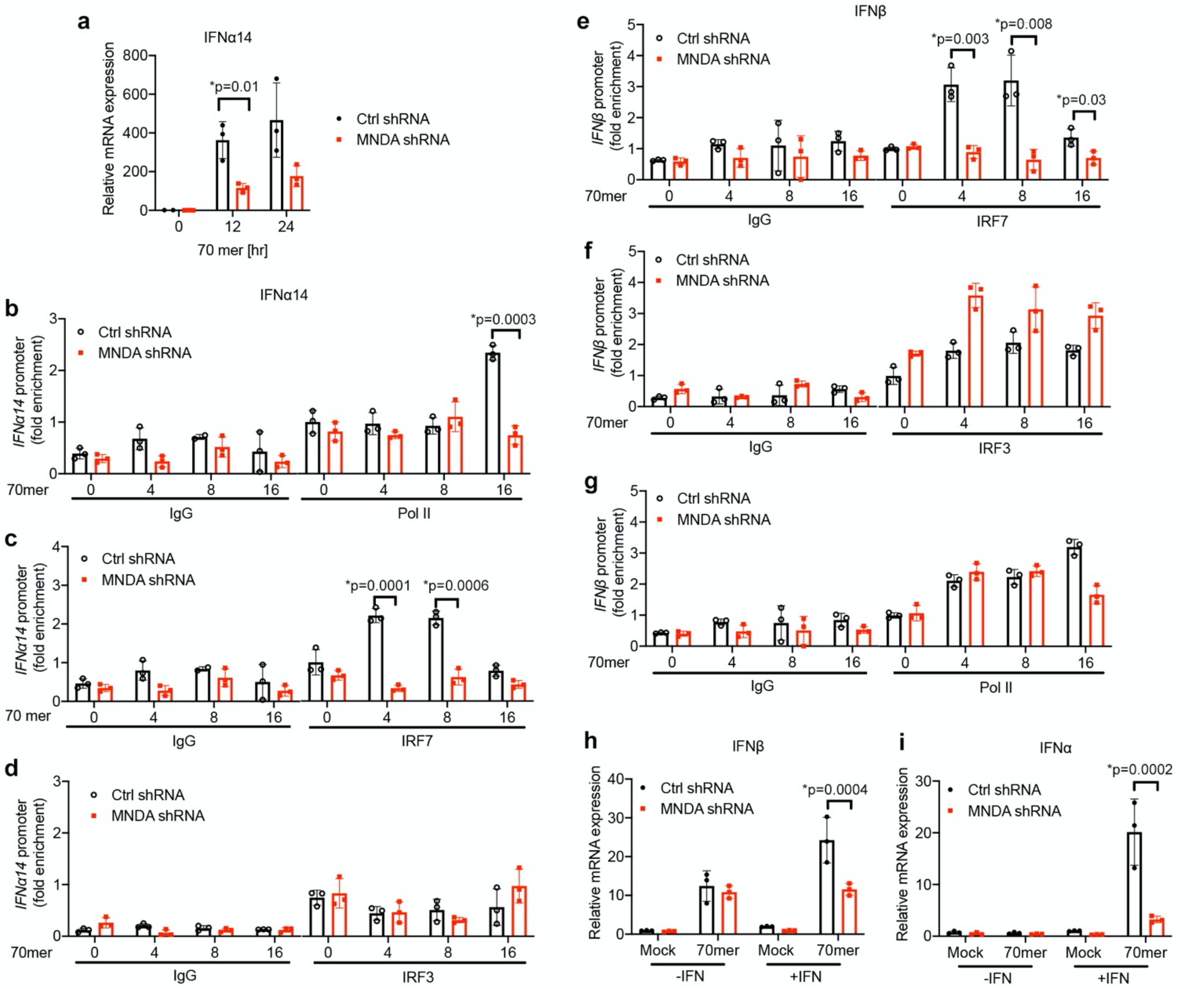

Supplementary Figure 7. Regulation of IRF7-dependent type I IFN promoter induction by MNDA. (a) Quantitative PCR analysis of IFNα14 mRNA in THP-1 cells expressing control or MNDA shRNA and transfected with dsVACV 70mer (1 µg/ml) for the indicated times. (b-d) Chromatin immunoprecipitation (ChIP) analysis of the recruitment of RNA Pol II (b), IRF7 (c) and IRF3 (d) to the IFNα14 promoter in THP-1 cells stably expressing control or MNDA shRNA and transfected with dsVACV 70mer (1 µg/ml) for the indicated times. Sheared chromatin lysates were subjected to ChIP with isotype control (IgG), and anti-RNA Pol II (b), anti-IRF7 (c), or anti-IRF3 (d) antibodies. (e-g) Chromatin immunoprecipitation (ChIP) analysis of the recruitment of IRF7 (e), IRF3 (f) and RNA Pol II (g) to the IFNβ promoter in THP-1 cells stably expressing control or MNDA shRNA, and transfected with dsVACV 70mer (1 µg/ml) for the indicated times. Sheared chromatin lysates were subjected to ChIP with isotype control (IgG), and anti-IRF7 (e), anti-IRF3 (f) or anti-RNA Pol II (g) antibodies. (h, i) Quantitative PCR analysis of IFNβ (h) IFNα (i) mRNA from THP-1 cells expressing control or MNDA shRNA, either left untreated or primed with IFNα for 16 h, before mock transfection or transfection with dsVACV 70mer (1 µg/ml) for a further 6 h. Data are mean ± SD of triplicate samples (a, h-k) or PCR technical triplicates (b-g) and are representative of three independent experiments; two tailed unpaired Students *t*-test; *p<0.05 indicates significance compared to respective groups.

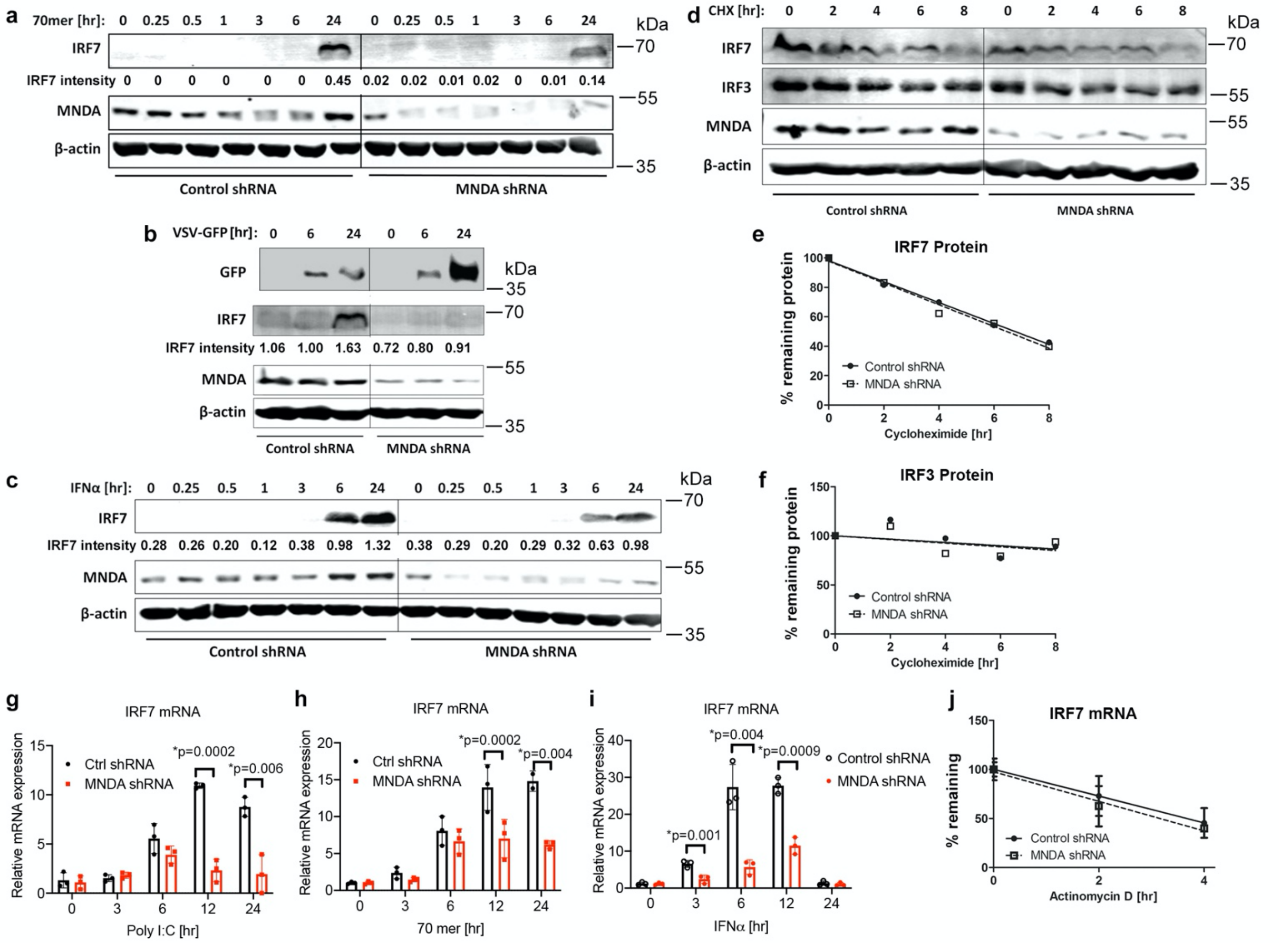

Supplementary Figure 8. MNDA-dependent upregulation of IRF7 protein is not due to regulation of stability of IRF7 protein or mRNA. (a) Immunoblot analysis of IRF7 and MNDA protein expression in THP-1 cells expressing control or MNDA shRNA transfected with dsVACV 70mer (1 µg/ml) for the indicated times. (b) Immunoblot analysis of GFP, IRF7 and MNDA protein expression in THP-1 cells expressing control or MNDA shRNA infected with VSV-GFP (MOI of 1) for the indicated times. (c) Immunoblot analysis of IRF7 and MNDA protein expression in THP-1 cells expressing control or MNDA shRNA treated with IFNα (1000 U/ml) for the indicated times. Numbers below IRF7 lanes (IRF7 intensity) are arbitrary units representing IRF7 band intensities, calculated relative to β-actin using ImageJ densitometric analysis. Representative of three experiments (a-c). (d-f) THP-1 cells stably expressing control or MNDA shRNA were treated with IFNα (1000 U/ml) for 16 h. Cells were further treated with 25µg/ml cycloheximide (CHX) for the indicated times. Lysates were prepared and immunoblotted for the indicated proteins (d) and IRF7 (e) and IRF3 (f) band intensities, relative to β-actin, were quantified by densitometry and expressed as a percentage of protein present in the absence of CHX. Representative of three experiments. (g-i) Quantitative PCR analysis of IRF7 mRNA from THP-1 cells expressing control or MNDA shRNA and transfected with poly(I:C) (2.5 ug/ml, g) or dsVACV 70mer (1 µg/ml, h) or treated with IFNα (1000 U/ml, i) for the indicated times. (j) Analysis of IRF7 mRNA stability. Quantitative PCR analysis of IRF7 mRNA from THP-1 cells expressing control or MNDA shRNA and treated with IFNα (1000 U/ml) for 16 h prior to treatment with actinomycin D (1 µg/ml) for the indicated times. Data are mean ± SD of triplicate samples (g-j) and are representative of three independent experiments; two tailed unpaired Students *t*-test; *p<0.05 indicates significance compared to respective groups.

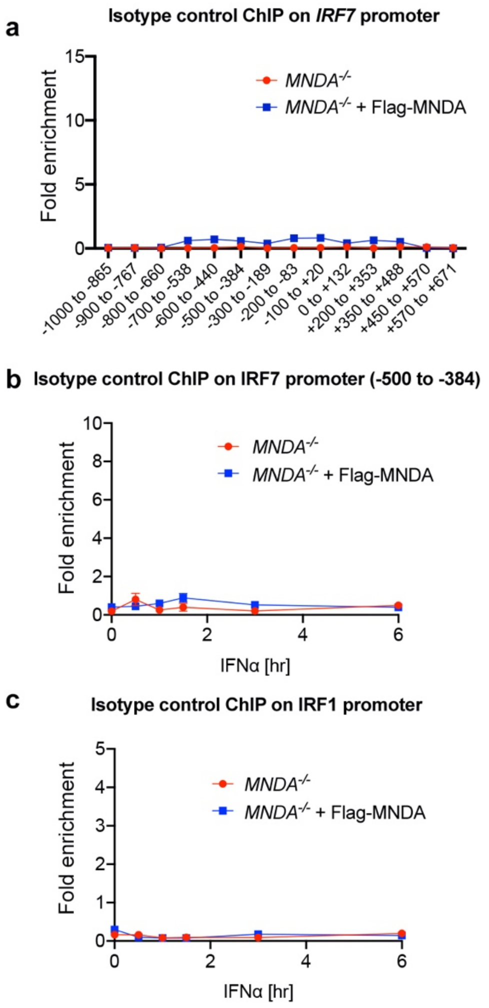

Supplementary Figure 9. Isotype controls for ChIP experiments in Figures 6 and 7. (a) Isotype control (IgG) for ChIP of the IRF7 promoter between the region of nucleotides -1000 to +671, using primer sets to amplify the specific regions indicated, in *MNDA*$^{-/-}$ cells expressing empty lentiviral vector or vector encoding Flag-MNDA. Cells were stimulated with IFNα (1000 U/ml) for 1 hr. (b, c) Isotype control (IgG) for ChIP of -500 to -384 region of the IRF7 promoter (b) or the IRF1 promoter (c) in *MNDA*$^{-/-}$ cells expressing empty lentiviral vector or vector encoding Flag-MNDA. Cells were treated with IFNα (1000 U/ml) for the indicated times. Data are presented as mean ± SEM of three independent experiments, each done with technical duplicates.

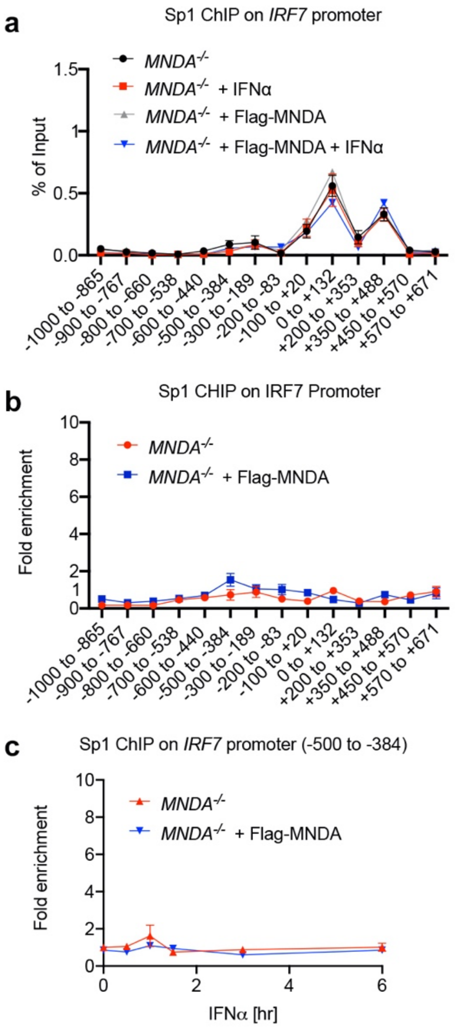

Supplementary Figure 10. No role for Sp1 in MNDA-dependent regulation of the IRF7 promoter. (a, b) ChIP analysis of Sp1 on the IRF7 promoter between the region of nucleotides -1000 to +671, using primer sets to amplify the specific regions indicated, in *MNDA*$^{-/-}$ cells expressing empty lentiviral vector or vector encoding Flag-MNDA. Cells were stimulated with IFNα (1000 U/ml) for 1 hr. Data is shown as both percent of input (a) and fold change compared to non-stimulated cells (b). (c) Sp1 ChIP of -500 to -384 region of the IRF7 promoter in *MNDA*$^{-/-}$ cells expressing empty lentiviral vector or vector encoding Flag-MNDA. Cells were treated with IFNα (1000 U/ml) for the indicated times. Data are presented as mean ± SEM of three independent experiments, each done with technical duplicates.
