# Supplementary material for: Myeloid cell nuclear differentiation antigen controls the pathogen-stimulated type I interferon cascade in human monocytes by transcriptional regulation of IRF7
Source: Nat Commun. 2022 Jan 10;13:14. doi: 10.1038/s41467-021-27701-x (PMC8748983; doi:10.1038/s41467-021-27701-x)
Supplement: Supplementary file 3 — Reporting summary [file 41467_2021_27701_MOESM3_ESM.pdf]

nature research

Corresponding author(s): Andrew Bowie

Last updated by author(s): Nov 19, 2021

Reporting Summary

Nature Research wishes to improve the reproducibility of the work that we publish. This form provides structure for consistency and transparency in reporting. For further information on Nature Research policies, see our Editorial Policies and the Editorial Policy Checklist.

Statistics

For all statistical analyses, confirm that the following items are present in the figure legend, table legend, main text, or Methods section.

n/a	Confirmed	
☐	☒	The exact sample size (n) for each experimental group/condition, given as a discrete number and unit of measurement
☒	☐	A statement on whether measurements were taken from distinct samples or whether the same sample was measured repeatedly
☐	☒	The statistical test(s) used AND whether they are one- or two-sided<br>*Only common tests should be described solely by name; describe more complex techniques in the Methods section.*
☒	☐	A description of all covariates tested
☒	☐	A description of any assumptions or corrections, such as tests of normality and adjustment for multiple comparisons
☐	☒	A full description of the statistical parameters including central tendency (e.g. means) or other basic estimates (e.g. regression coefficient) AND variation (e.g. standard deviation) or associated estimates of uncertainty (e.g. confidence intervals)
☐	☒	For null hypothesis testing, the test statistic (e.g. F, t, r) with confidence intervals, effect sizes, degrees of freedom and P value noted<br>*Give P values as exact values whenever suitable.*
☒	☐	For Bayesian analysis, information on the choice of priors and Markov chain Monte Carlo settings
☒	☐	For hierarchical and complex designs, identification of the appropriate level for tests and full reporting of outcomes
☒	☐	Estimates of effect sizes (e.g. Cohen's d, Pearson's r), indicating how they were calculated

Our web collection on statistics for biologists contains articles on many of the points above.

Software and code

Policy information about availability of computer code

Data collection	No software was used
Data analysis	FlowJo software version 10 (TreeStar) was used to analyse flow cytometry data. ImageJ software version 1.42 was used for image acquisition and densitometric analysis of immunoblots

For manuscripts utilizing custom algorithms or software that are central to the research but not yet described in published literature, software must be made available to editors and reviewers. We strongly encourage code deposition in a community repository (e.g. GitHub). See the Nature Research guidelines for submitting code & software for further information.

Data

Policy information about availability of data

All manuscripts must include a data availability statement. This statement should provide the following information, where applicable:

- Accession codes, unique identifiers, or web links for publicly available datasets
- A list of figures that have associated raw data
- A description of any restrictions on data availability

All data generated or analysed during this study are included within this article (and its supplementary information files). Source data are provided with this paper.

Field-specific reporting

Life sciences study design

All studies must disclose on these points even when the disclosure is negative.

Sample size	Samples are mainly cells grown in culture and as is standard in this field of research, samples were normally set up in triplicate, For data involving human PBMCs in Fig 1I-K, six donors were used.<br><br>We used a sample size of three replicates per treatment, based on our previous experience with similar experiments (PMID 32102850, 31558608, 31076360). The exception was for ChIP assays where due to the scale of the experiment, technical duplicates rather than triplicates were used. For experiments analysing responses in primary human monocytes (Fig 1h-l) we increased the sample size to six to increase the statistical power and account for the greter biological variation of primary cells from separate donors.
Data exclusions	No data were excluded
Replication	All data shown, unless stated below in legends, is representative of three independent experiments, and all attempts at replication were successful.
Randomization	Cells were assigned into groups according to genotype of interest (siRNA- or shRNA-treated or CRISPR/Cas9-generated), and thereafter the selection of which cells received what treatment was random
Blinding	Blinding was not meaningful as the same investigator performed the experiments and analyzed the data.

Reporting for specific materials, systems and methods

We require information from authors about some types of materials, experimental systems and methods used in many studies. Here, indicate whether each material, system or method listed is relevant to your study. If you are not sure if a list item applies to your research, read the appropriate section before selecting a response.

Materials & experimental systems

n/a	Involved in the study	
☐	☒	Antibodies
☐	☒	Eukaryotic cell lines
☒	☐	Palaeontology and archaeology
☒	☐	Animals and other organisms
☐	☒	Human research participants
☒	☐	Clinical data
☒	☐	Dual use research of concern

Methods

n/a	Involved in the study	
☒	☐	ChIP-seq
☐	☒	Flow cytometry
☒	☐	MRI-based neuroimaging

Antibodies

Antibodies used

Primary antibodies used were:
Anti-MNDA (Cell Signaling, 3C1, #3329),
Anti-phospho-IRF3 (Cell Signaling, 4D4G, #4947),
Anti- IRF7 (Cell Signaling, rabbit polyclonal, #4920S),
Anti-phospho-Y701-STAT1 (Cell Signaling, rabbit polyclonal, #9171S)
Anti-STAT1 (Cell Signaling, rabbit polyclonal, #9172S)
Anti-IRF3 (Immuno-Biological Laboratories, rabbit polyclonal, #18781)
Anti-IFI16 (Santa Cruz, 1G7, #sc-8023)
Anti-GFP (Santa Cruz, B-2, #sc-9996)
Anti-Lamin A/C (Santa Cruz, 636, #sc-7292)
Anti-AIM2 (Adipogen, 3B10, #AG-20B-0040)
Anti-β-actin (Sigma, AC-74, #A5316)
Anti-tubulin (Millipore, DM1A, #MABT205)
Anti-PYHIN1 (from Jin-Fong Lee (University of Texas, USA), rabbit polyclonal, described in PMID: 16479015)
Anti-IFIT3 (from Andreas Pichlmair (Technical University of Munich, Germany), rabbit polyclonal, described in PMID: 21642987)
Anti-Flag (Sigma, M2, #F3165)
Anti-CD14 APC (eBiosciences, 61D3, #17-0149-42)
anti-CD19 PE (eBiosciences, HIB19, #12-0199-42)
anti CD3 PE-Cy5.5 (eBiosciences, SK7, #35-0036-42)
anti-CD56 PE-Cy7 (eBiosciences, CMSSB, #25-0567-42)
Anti-Pol II (Santa Cruz, N-20 X, #sc-899),
Anti-IRF7 (Santa Cruz, H-246 X, #sc-9083)
Anti-IRF3 (Santa Cruz, FL-425 X, #sc-9082)
Anti-Sp1 (Abcam, rabbit polyclonal, #ab13370)

	Anti-STAT2 (Santa Cruz, B-3, #sc-514193)<br>Anti-Histone H4K5,8,12,16ac (Merck, rabbit polyclonal, #06-866)
Validation	For commercially purchased antibodies, all antibodies were validated by the supplier. For all antibodies purchased, apart from those from eBiosciences which were used for cell sorting in flow cytometry, a dataset was provided showing a western blot that detected the protein at the expected size. Further, all antibodies we used for immunoblotting detected the protein of expected size, as shown for the full blots in the source data file, including the IFIT3 and PYHIN1 antibody which were provided by collaborators. Also, for antibodies to MNDA, IRF7, IFI16, PYHIN1 and AIM2, siRNA or CRISPR/Cas9 experiments further validated that these antibodies were detected the correct protien. Flow cytometry antibodies are standardly used to label different subsets of cells and representative FACS plots using each antibody are shown in Supplemental Figures. ChIP antibodies were used in conjunction with the appropriate isotype control (shown in Supplementary Figures).

Eukaryotic cell lines

Policy information about cell lines

Cell line source(s)	THP-1, HEK293 and HEK293T cells were purchased from the European Collection of Cell Cultures. HEK-Blue IFN-α/β reporter cells were purchased from InvivoGen. The human pDC cell line CAL-1 were a gift from Dr T. Maeda, Department of Island and Community Medicine, Nagasaki University, Japan. The establishment of CAL-1 cells from a patient with blastic NK cell lymphoma is described in PMID: 15765784
Authentication	The cell lines have been extensively used in our lab, but not formally authenticated
Mycoplasma contamination	THP-1, HEK293 and HEK293T tested negative for mycoplasma. Other cell lines used were not tested for mycoplasma.
Commonly misidentified lines<br>(See ICLAC register)	None

Human research participants

Policy information about studies involving human research participants

Population characteristics	PBMCs were obtained from anonymous healthy donors in the Irish population who had given blood to the Irish Blood Transfusion Service.
Recruitment	Donors provide written agreement that their blood can be used for research purposes. As the donors were anonymous to us, there is no bias.
Ethics oversight	School of Biochemistry and Immunology Research Ethics Committee, Trinity College Dublin

Note that full information on the approval of the study protocol must also be provided in the manuscript.

Flow Cytometry

Plots

Confirm that:

- [x] The axis labels state the marker and fluorochrome used (e.g. CD4-FITC).
- [x] The axis scales are clearly visible. Include numbers along axes only for bottom left plot of group (a 'group' is an analysis of identical markers).
- [x] All plots are contour plots with outliers or pseudocolor plots.
- [x] A numerical value for number of cells or percentage (with statistics) is provided.

Methodology

Sample preparation	PBMCs were stained with antibodies to identify sub-populations of cells. THP-1 cells were infected for 48 h with VSV-GFP.
Instrument	FACSCanto II Flow Cytometer (BD Biosciences)
Software	FlowJo software (TreeStar)
Cell population abundance	A pure population of THP-1 cells only was used and analysed for GFP-positive cells
Gating strategy	Gating strategies are shown in Supplemental Figures 1 and 4.

- [x] Tick this box to confirm that a figure exemplifying the gating strategy is provided in the Supplementary Information.
